# Supplementary material for: Regional Gray Matter Volume Changes in Parkinson’s Disease with Orthostatic Hypotension
Source: Brain Sci. 2021 Feb 26;11(3):294. doi: 10.3390/brainsci11030294 (PMC7996881; doi:10.3390/brainsci11030294)
Supplement: Supplementary file 1 [file brainsci-11-00294-s001.pdf]

Supplementary Table. Qualitative scoring method for the pentagon copying test (QSPT).

Parameters	Performance scores	Assigned scores
1. Number of angles	10	4
	10 ± 1	3
	10 ± 2	2
	5-7	1
	<5 or >13	0
2. Distance/Intersection	Correct intersection	4
	Wrong intersection	3
	Contact without intersection	2
	No contact, distance < 1cm	1
	No contact, distance > 1cm	0
3. Closure/opening*	Closing both figures	2
	Closing only one figure	1
	Opening both figures	0
4. Rotation**	Correct orientation of both figures	2
	Rotation of one figure (either one figure is absent or it is not a pentagon then it is not assessable)	1
	Rotation of both figures (or both not assessable like pentagons)	0
5. Closing-in	Absent	1
	Present	0
Total	Sum of 1-5	0-13

*Figure is considered close even though two sides do not touch each other, but the distance is ≤ 1 mm.

**When there is not a figure or figure is not a pentagon (then rotation is not assessable) score is 0. When rotation is less than 45°, figure is not considered rotated. Tremor is ignored.
